# Comparisons of the biodistribution and toxicological examinations after repeated intravenous administration of silver and gold nanoparticles in mice

**DOI:** 10.1038/s41598-017-03015-1

**Published:** 2017-06-12

**Authors:** Lin Yang, Huijuan Kuang, Wanyi Zhang, Zoraida P. Aguilar, Hua Wei, Hengyi Xu

**Affiliations:** 1 State Key Laboratory of Food Science and Technology, Nanchang University, Nanchang, 330047 China; 2 The Second Affiliated Hospital of Nanchang University, Nanchang, 330000 China; 3 Zystein, LLC., Fayetteville, AR 72704 USA

## Abstract

Nanoparticles (NPs) size, surface functionalization, and concentration were claimed to contribute to distribution and toxicity outcomes of NPs *in vivo*. However, intrinsic chemical compositions of NPs caused inconsistent biodistribution and toxic profiles which attracted little attention. In this study, silver nanoparticles (AgNPs) and gold nanoparticles (AuNPs) were used to determine the biodistribution, toxickinetic, and genotoxicity variances in murine animals. The results demonstrated AgNPs and AuNPs were primarily deposited in the mononuclear phagocyte system (MPS) such as the liver and spleen. In particular, AuNPs seemed to be prominently stored in the liver, whereas AgNPs preferentially accumulated in more organs such as the heart, lung, kidney, etc. Also, the circulation in the blood and fecal excretions showed higher AgNPs contents in comparison with the AuNPs. Measurements of the mouse body and organ mass, hematology and biochemistry evaluation, and histopathological examinations indicated slight toxic difference between the AgNPs and AuNPs over a period of two months. RT-qPCR data revealed that AgNPs induced greater changes in gene expression with relevance to oxidative stress, apoptosis, and ion transport. Our observations proved that the NPs chemical composition played a critical role in their *in vivo* biodistribution and toxicity.

## Introduction

Inorganic nanoparticles (NPs) have attracted increasing attention and are used in many fields, such as biomedicine, various industries, and electronics due to their excellent physicochemical properties^1, 2^. Currently, silver nanoparticles (AgNPs) are one of the most widely used NPs in commercial products (*e*.*g*. wound dressings, contraceptive devices, and packaging materials) because of their strong antimicrobial and anti-inflammatory properties^2, 3^. Gold nanoparticles (AuNPs) offer a wide range of applications including cosmetics, chemical sensing, drug carriers, bioimaging, and gene therapy^4, 5^. The extended use of these inorganic NPs can lead to frequent human exposure via various routes (*e*.*g*. ingestion, inhalation and dermal contact) during manufacture, use and disposal. Moreover, compared with their bulk counterparts, AgNPs and AuNPs hold greater surface area to volume ratio, higher particle reactivity, and can undergo surface modifications which could result in greater side effects once these are absorbed into the body^6^. It is therefore imperative to understand the potential biological responses to AgNPs and AuNPs *in vivo*.

Indeed, previous *in vitro* studies demonstrated that inorganic NPs can induce the production of reactive oxygen species (ROS), DNA damage, and apoptosis^7–10^. These observations resulted in a number of *in vivo* rodent studies that were focused on the toxicity or biokinetics of inorganic NPs following various administration routes^6, 11–33^. Table S1 summarized the biodistribution and/or toxicity profile of various types of AgNPs and AuNPs in rodents following oral, instillation, inhalation, and intraperitoneal exposures. Prolonged inhalational exposure to AgNPs induced lung function changes and inflammatory responses^12^. Major organs of accumulation for AgNPs have been shown to be the lungs and liver in a 90-day inhalation toxicity study^23^, and liver in several long term oral exposure studies^20, 22, 24^. Also, intratracheal injection showed that the majority of AuNPs were found in the lungs regardless of PEG surface modification^25^. Of note, during oral and inhalation exposures, or dermal contact, a few NPs were able to pass through the gastrointestinal tract (GIT), lung barriers, and dermis into the blood, resulted in extremely low amount of NPs translocated to the secondary organs and thus difficult to detect and compare the accumulation profile of the NPs in the whole body^22, 28^. To avoid limited systemic exposure due to the cellular barriers present in the GIT, lung, and skin, the present study was used to repeated intravenous administration of two inorganic NPs to compare their toxicity and distribution profiles in mice.

The classical *in vivo* studies in rodents through intravenous injection, which examined the distribution and/or toxicity of various types of AgNPs and AuNPs with sizes ranging from 1.4 nm to 250 nm were enumerated in Table 1. These studies showed that the NPs size, shape, dose, and surface modifications are responsible for the difference in toxicity and/or accumulation of NPs. Dose-dependent toxicity was observed following exposure to four different concentrations (4, 10, 20, 40 mg/kg) of intravenously administered AgNPs^19^. Meanwhile, they also exhibited that the major accumulation organs were observed in the liver and spleen, and the smaller NPs had a more widespread organ distribution^13, 26, 27^. More specifically, 10 nm AuNPs were found in various organs including the liver, spleen, kidney, heart, lungs, testis, brain, and thymus, whereas 250 nm AuNPs were restricted to the liver and spleen^26^. These studies have reported strong influences of exposure routes, animal gender, size, shape, concentration, surface coating, aggregation, stability, or purities of nanomaterials on the biodistribution of AgNPs and AuNPs^10, 13, 14, 20–22^.

**Table 1:** Summary of biodistribution or toxicity of AuNPs/AgNPs in rodents via exposure of intravenous injection.

NPs	Size (nm)	Dose	Model	Exposure duration	Tissue accumulation (in order of quantity)	Conclusions	References
AgNPs	20, 200	5 mg kg^−1^	Rats	1, 7, 28 d	Li, Sp, Ki, Lu, Br, Ur, Fe	Higher tissue burden in 20 nm AgNPs group compared with 200 nm NPs group	2
AuNPs	20	0.01 mg kg^−1^	Rats	1, 7 d, 1, 2 m	Li, Sp, Lu, Ki, He, Ur, Fe (Over 25 organs)	AuNPs widely deposited in the body over 2 months and caused gene expression changes after a single exposure	6
AgNPs	7.2 ± 3.3	45, 5, 10 mg kg^−1^	Rats	1, 3 d	NM	Body weight and locomotor activity were decreased	11
AuNPs	1.4, 5, 18, 80, 200, 2.8	1.6 ± 0.2–43.7 ± 5.3 μg/rat	Rats	1 d	Li, Sp, Ki, Ca, Lu, Bl, GIT, Ut, He, Br,	Size and surface charge dependent distribution of NPs, *e*.*g*. most NPs accumulated in the liver increased from 50% of 1.4 nm NPs to >99% of 200 nm NPs	13
Au nanorods	NM	0.56 mg kg^−1^	Mice	4 h, 1 d	Li, Sp, Lu, Ki, He, Tu	Gold nanorods reached to tumor tissue and had low toxicity, which was related to the surface modifications of NPs	17
AgNPs	15–40	4, 10, 20, 40 mg kg^−1^	Rats	32 d	Li, Ki	AgNPs with dose <10 mg kg^−1^ is safe, while it is toxic when a dose over 20 mg kg^−1^	19
AgNPs	21.8	7.5, 30, 120 mg kg^−1^	Mice	1, 7, 14 d	Sp, Li, Lu, Ki	AgNPs could be deposited primarily in liver and spleen as well as other tissues. The circulation and elimination of NPs showed gender-related difference	21
AuNPs (PEGylated)	11, 21, 31	0.07–0.30 mg kg^−1^	Rats	1 h, 1 d	Li, Sp, Ca, Bl, GIT + Fe, Lu, Ki, He, Ur, Br	10 kDa PEG modified NPs showed prolonged blood circulation time, compared to non-PEGylated and 750 Da PEGylated AuNPs	25
AuNPs	10, 50, 100, 250	0.077–0.108 mg kg^−1^	Rats	1 d	Li, Sp, Bl, Lu, Ki, He, Thy, Br, Te	Size-dependent tissue distribution of AuNPs with the smallest 10 nm showing the most widespread organ distribution	26
AuNPs	15, 50, 100, 200	1000 mg kg^−1^	Mice	1 d	Li, Lu, Ki, Sp, He, Br, Bl, St, Pa	Size-dependent biodistribution of AuNPs with only smaller NPs (15 and 50 nm) crossing the blood-brain barrier	27
AgNPs	20, 100	0.07–6 mg kg^−1^	Rats	28 d	NM	Immune system is the most sensitive parameter that could be affected by AgNPs, *e*. *g*. a dose of 0.01 mg kg^−1^ NPs decreased thymus weight	28
AgNPs	35.3 ± 8.2	0.5 mg kg^−1^	Rats	1, 3, 5 d	Li, Sp, Lu, Ki, Br, Bl	Toxicity of AgNPs is mainly due to its intact nanostructure with minor contributions from its released silver ions	30
AuNPs	13 ± 1	0.004–0.04 mg kg^−1^	Rats	1 h, 1 d	Li, Sp, Ca, Bl, Ki, GIT + Fe, Lu, Ut, He, Ur, Br	Polymer coated AuNPs with high colloidal stability can be degraded *in vivo* that caused by proteolytic enzymes	31
AuNPs	16.1	0.7 mg kg^−1^	Rats	0.5 h, 28 d	Li, Sp, He, Ta, Lu, Bo, Bl, Ki, In, Te, Thy, Br, Mu	Surface coating showed greater effects on toxicity instead of on biodistribution of the AuNPs	32
AuNPs	1.4, 80	0.011, 0.11 mg kg^−1^	Rats	1 d	Li, Ca, Ur, Sk, Ki, GIT + Fe, Bl, Sp, Lu, Ut, He, Br	The accumulation of 18 nm in spleen and liver is significantly higher compared with the 1.4 nm AuNPs	33
AgNPs, AuNPs	3 ± 1.57, 6	11.4–13.3 mg kg^−1^	Mice	28, 56 d	Li, Sp, Ki, He, Lu, Te, Fe, Bl, In, St, Br, SV	AuNPs were mainly stored in the liver, whereas AgNPs were widely stored in more organs including the lung, brain, testis, etc	This study

The abbreviations are used in the Table: NM – not mentioned, h – hour, d – day, w – week, m – month, Br – brain, He – heart, Li – liver, Lu – lung, Ki – kidney, In – intestine, Sk – skin, Sp – spleen, GIT – gastro-intestinal tract, Fe – feces, St – stomach, Te – testes, Thy – thymus, Tu – tumor, Ut – uterus, Ur – urine, Ca – carcass, Bl – blood, Bo – bones, SV – seminal vesicle, Mu – muscle, Pa –pancreas, Ta – tail.

However, it is still unclear whether distribution, translocation, and elimination of AgNPs and AuNPs would mainly result from the NPs size effects or NPs chemical composition (such as released the free ions). In a 28-day oral exposure study, silver acetate (Ag ions) showed higher organ distribution throughout the rat body compared with AgNPs^29^. Also, it was demonstrated that the silver concentration in organs was closely related with the Ag^+^ concentration in the AgNPs suspension which could be used to deduce that only silver ions passed through the GIT causing accumulation in the liver and spleen^3^. Both studies exhibited that the released silver ions and not the “nano-size” affected the NPs distribution and toxicity after oral exposure. Additionally, AgNPs and AuNPs were used to study whether the toxicity induced by NPs size or NPs chemical composition in a zebrafish model^34^, because both NPs possess similar chemical characteristics (water insoluble, stable, and rarely present in biological tissues). Nevertheless, no rodent toxicity studies have shown possible biodistribution and/or toxicity profiles among NPs with different chemical compositions and identical surface coating such as AgNPs and AuNPs after intravenous administration, even though the NPs chemical composition, as well as particle size, exposure route, and surface chemistry, is an important parameter that contributed to the toxicological responses.

The aim of the present study was to determine the differential accumulation, distribution, toxicity, and potential genotoxicity of AgNPs and AuNPs in mice following repeated intravenous injection for 4 weeks, at a dose with limited systemic toxicity as reported in a relevant study^19^. Both carboxylic acid coated AgNPs and AuNPs in this study can be conjugated to peptide, protein, and other amine containing molecules for various biomedicine applications. Herein, as a function of chemical composition, the effects of AgNPs and AuNPs on toxicokinetic and toxicity were evaluated to understand their nanotoxicity.

## Results

### Characterization of AgNPs and AuNPs

**Figure 1:**
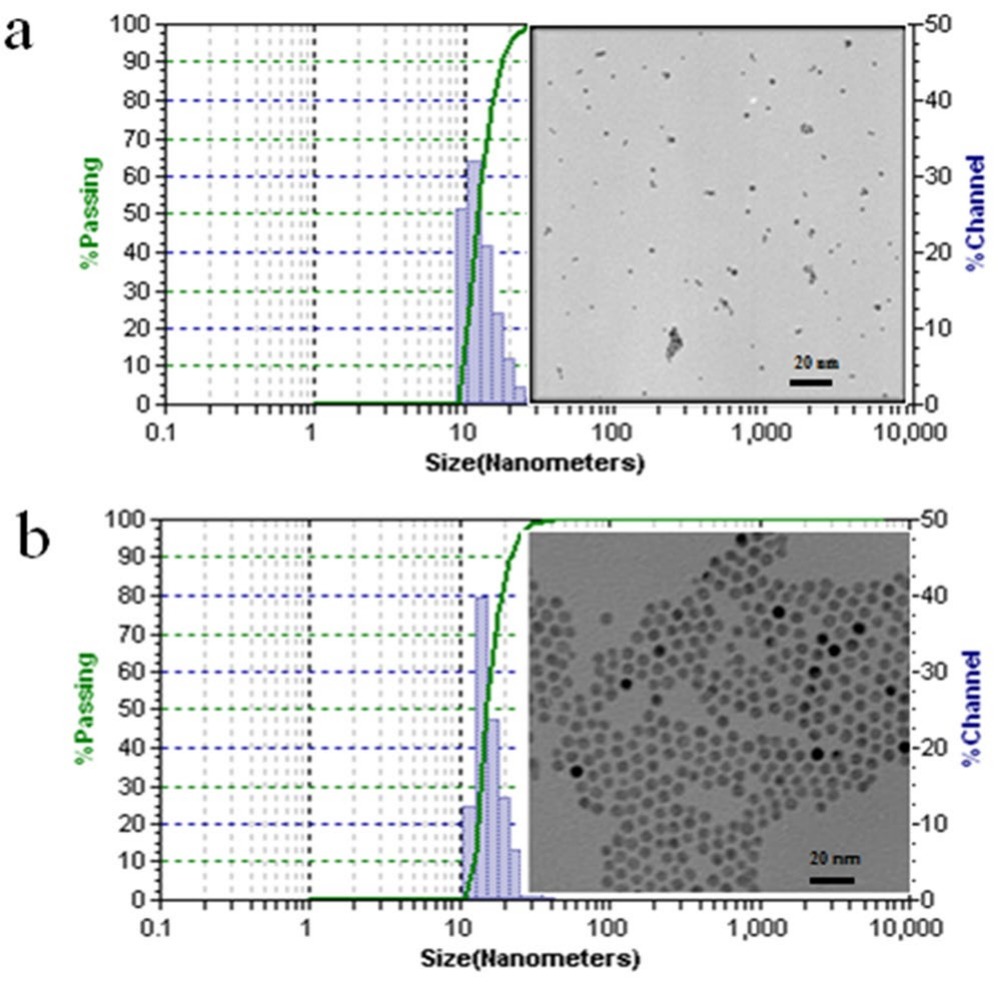
Characterizations of amphiphilic polymers coated AgNPs (**a**) and AuNPs (**b**) (TEM and DLS).

As shown in Fig. 1, the AuNPs exhibited uniform spherical and monodisperse state with core size of 6 nm, while the AgNPs showed uniform spherical but less monodisperse state with core size of 3 ± 1.57 nm (n = 4). The hydrodynamic diameter (HD) distribution of the inorganic NPs (shown in Fig. 1) were 12.22 ± 6.05 and 15.07 ± 6.68 nm, which were larger than the corresponding sizes from the transmission electron microscopy (TEM) measurements. Dynamic light scattering (DLS) measurements of inorganic NPs showed bigger diameter due to different surface states, water of hydration and agglomeration^3, 35^.

### Effects on body weight and organ index

**Figure 2:**
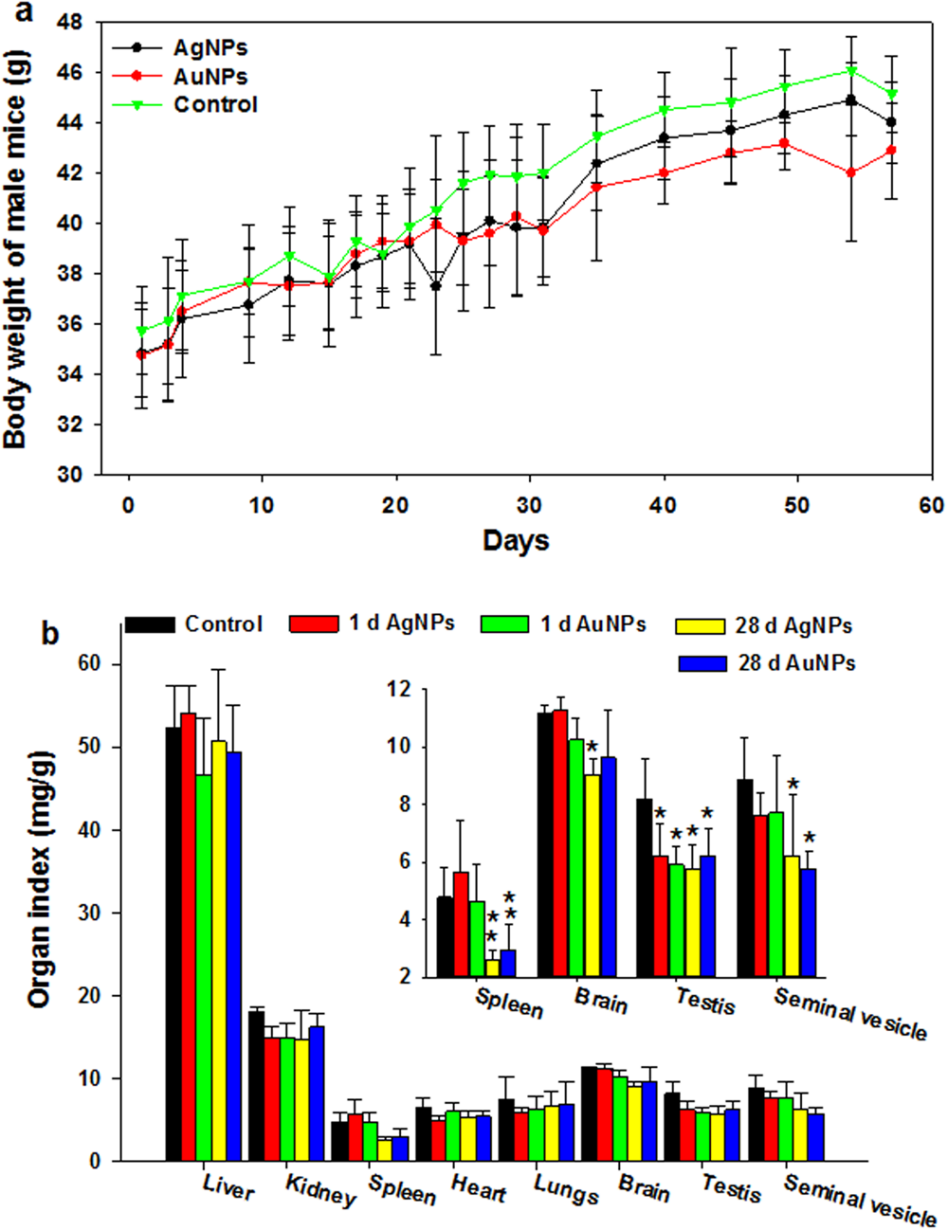
Body weight (**a**) and organ index (**b**) of Kunming mice following intravenous injection of AgNPs and/or AuNPs. All the administration doses were 11.4–13.3 mg/kg. n = 5.

The food consumption, body weight, and organ index of each animal during the entire experimental period were evaluated as previously reported^11, 20, 23, 28, 35–37^. No significant changes in food consumption and body weight were observed in either of the animal groups treated with AgNPs or AuNPs. As shown in Fig. 2a, the mouse body weight of two experimental groups and the control group displayed similar increasing trends. Also, the liver, kidney, heart, and lungs showed no significant differences in mass in comparison with the control. However, weights of spleen and seminal vesicle in both NPs groups were significantly lower than controls at 28 d post the last injection (dpi). Additionally, the brain exhibited significant weight decrease at 28 dpi in the AgNPs group, while the testis had significant weight decrease at both time points for both NPs (Fig. 2b and Table S2).

### Biodistribution and elimination of AgNPs and/or AuNPs

**Figure 3:**
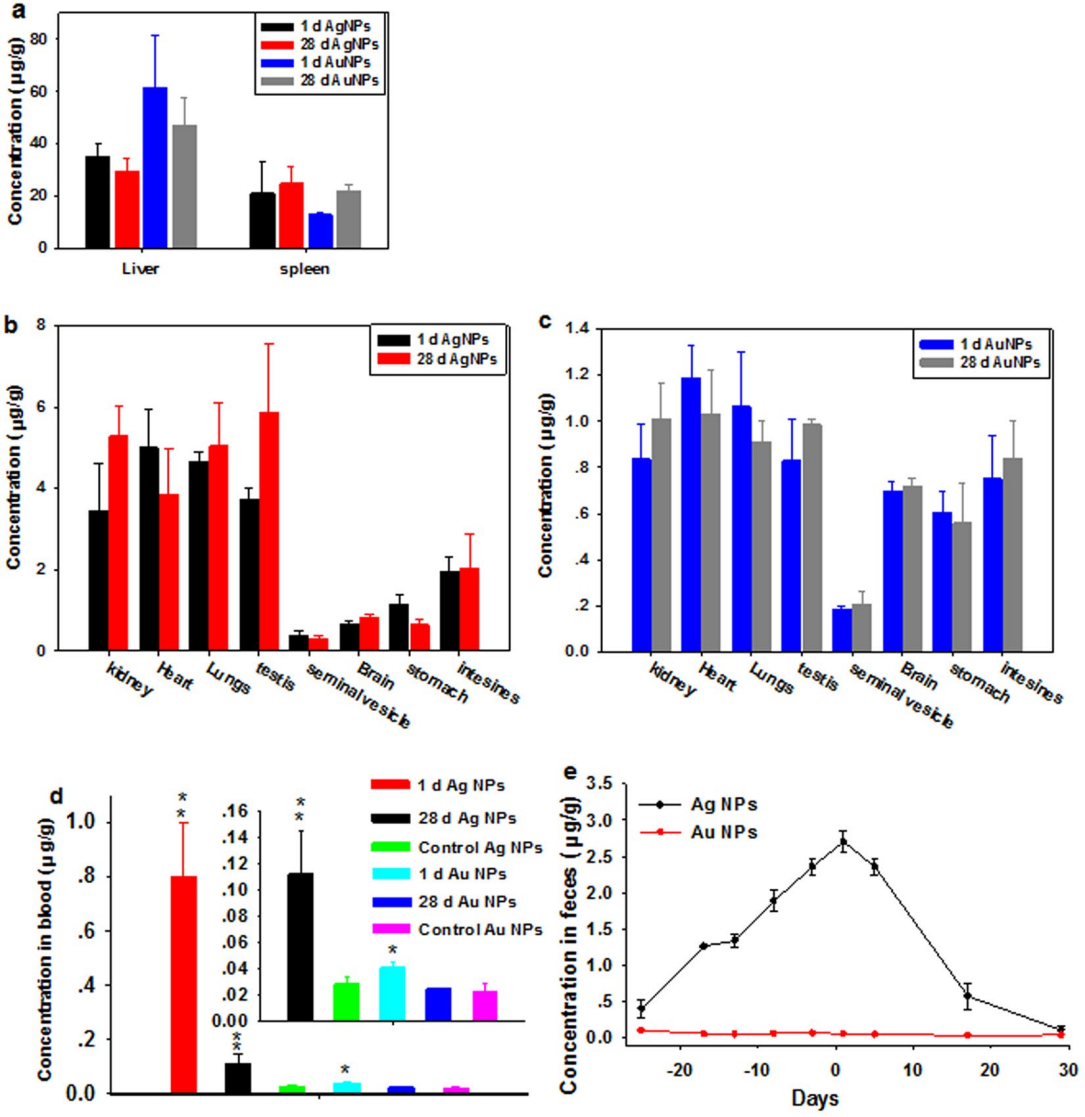
AgNPs or AuNPs levels in animal tissues (**a**–**c**), blood (**d**) and feces (**e**) as indicators of inorganic NPs biodistribution, circulation and elimination at serial time points. These results show mean and standard deviation, n = 5. **a** Exhibits the contents of AgNPs or AuNPs in the liver and spleen at both time points, **b** and **c** exhibit the contents of AgNPs or AuNPs in the other organs at both time points, respectively. Day −25 to day 0 are the time interval during which the NPs were administered.

As shown in Fig. 3, AgNPs and AuNPs were both accumulated in the liver and spleen, followed by the kidney, heart, lungs and testis, and the least accumulation was found in the stomach, intestine, and seminal vesicle at 1 dpi. Note that the AuNPs concentrations (61.39 ± 19.99, 47 ± 10.62 μg/g) were significantly higher than the AgNPs concentrations (35.06 ± 4.78, 29.69 ± 4.50 μg/g) in the liver at both time points after same dose exposure (Fig. 3a). However, the concentrations of AgNPs showed higher levels in the spleen at both time points compared with AuNPs (Fig. 3a). Furthermore, concentrations of AgNPs were approximately 4–5.5 times higher than the concentrations of AuNPs in the kidney, lung, heart, and testis (Fig. 3b,c and Table S3). For instance, in the kidney, the concentration of AgNPs was 3.44 ± 1.15 μg/g, which was about 4.14 times compared with the concentration of AuNPs (0.83 ± 0.14 μg/g) at 1 dpi.

The kinetics of AgNPs and/or AuNPs in the blood and feces was determined by measuring the AgNPs or AuNPs concentrations in successively collected samples. Figure 3d showed undetectable gold contents in blood at both time points, suggesting that AuNPs was rapidly cleared from the blood and effectively uptaken by MPS. By contrast, the concentration of AgNPs (0.80 ± 0.19 μg/g) in the blood exhibited higher concentration compared with the AuNPs (0.041 ± 0.004 μg/g) at 1 dpi, and then decreased over time. Figure 3e showed that the elimination rate of the AgNPs (12.22 ± 6.05 nm) in the feces of mice gradually increased after injection and reached a maximum value (2.70 ± 0.15 μg/g) at 1 dpi, and then decreased over time. But the AuNPs concentration in the feces could not be detected during the entire experimental period. Additionally, the total recovery ratios of applied AgNPs and/or AuNPs were 35.87 ± 9.94%, 31.17 ± 9.67%, and 42.54 ± 18.87%, 27.78 ± 6.13% at 1 dpi and 28 dpi, respectively (Table S4).

### Biochemistry and hematology results

**Figure 4:**
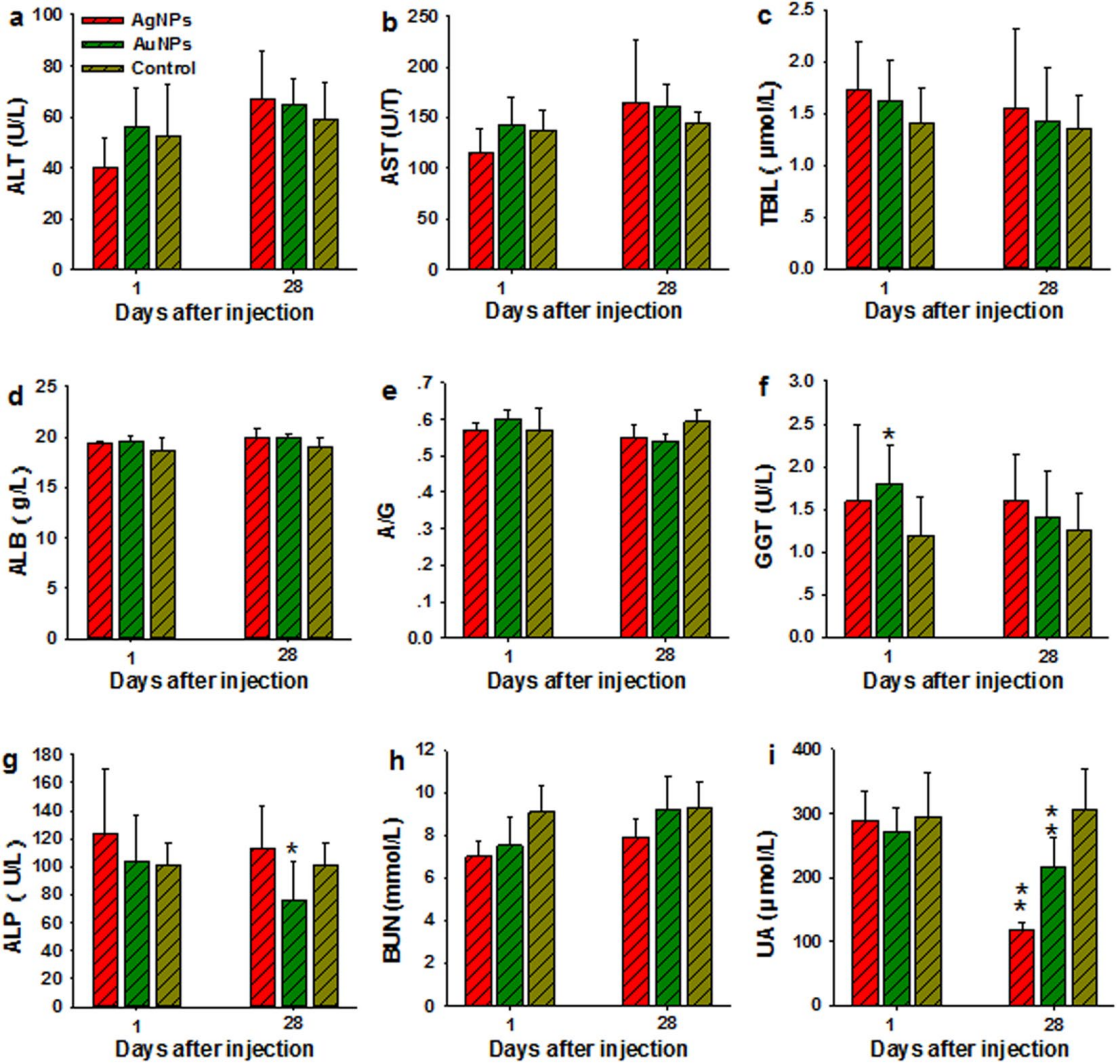
Serum biochemical analysis from animals treated with AgNPs and/or AuNPs and control. (**a–i**) Results exhibit mean and standard deviation of ALT (**a**), AST (**b**), TBIL (**c**), ALB (**d**), A/G (**e**), GGT (**f**), ALP (**g**), BUN (**h**), and UA (**i**). Abbreviations: alanine aminotransferase, ALT; aspartate aminotransferase, AST; total bilirubin, TBIL; albumin, ALB; the ratio of albumin to globulin, A/G; gamma glutamyl transaminase, GGT; alkaline phosphatase, ALP; blood urea nitrogen, BUN; and urea, UA, n = 5.

**Figure 5:**
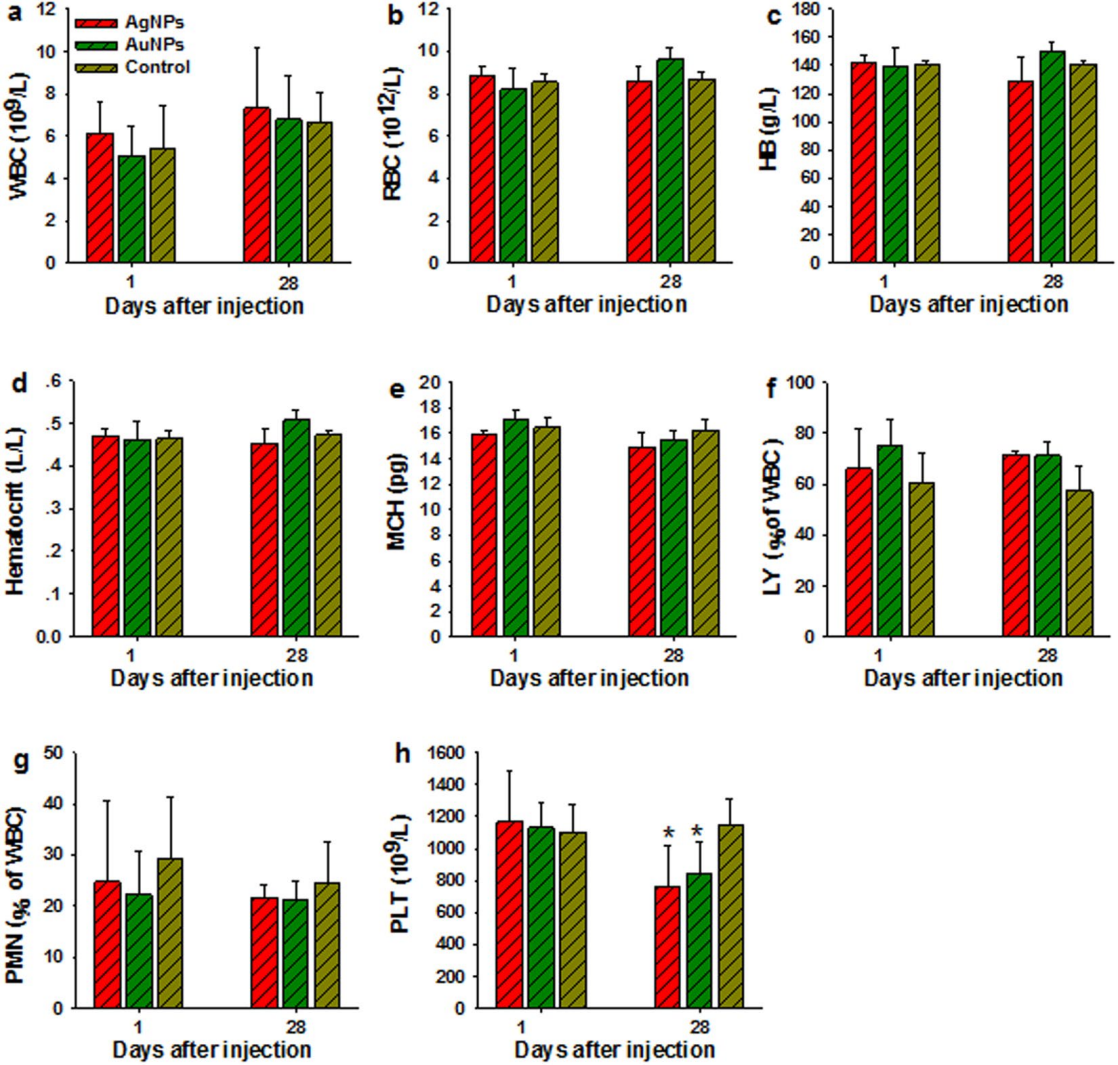
Whole blood analysis from animals treated with AgNPs and/or AuNPs and control. (**a–h**) Results exhibit mean and standard deviation of white blood cells count, WBC (**a**); red blood cells count, RBC (**b**); hemoglobin, HB (**c**); hematocrit (**d**); mean corpuscular hemoglobin, MCH (**e**), lymphocyte, LY (**f**); polymorphomultinuclear neutrophil granulocyte PMN (**g**), and platelet count, PLT (**h**), n = 5.

The inorganic NPs used here were similar in sizes to proteins or viruses which could induce inflammatory response, immune response, and alter related hematological factors in the blood such as white blood cell count^38^. As a result, we determined the standard inflammatory biochemical parameters such as alanine aminotransferase (ALT), aspartate aminotransferase (AST), total bilirubin (TBIL), albumin (ALB), the ratio of albumin to globulin (A/G), gamma glutamyl transaminase (GGT), alkaline phosphatase (ALP), blood urea nitrogen (BUN), and urea (UA). These biochemical markers, except the GGT, ALP, and UA, in either AgNPs or AuNPs treated groups were not statistically different from the control groups as shown in Fig. 4. The mice treated with AuNPs showed significantly increased level of GGT and decreased level of ALP (P < 0.05) but remained within the normal range at 1 dpi and 28 dpi, which may due to the highest uptake of AuNPs in the liver. In particular, UA as an indicator of renal function exhibited significantly reduced levels of AuNPs and AgNPs in all the groups, which need further investigation. Several important hematology markers including the white blood cell count (WBC), red blood cell count (RBC), hemoglobin (HB), mean corpuscular hemoglobin (MCH), polymorph multinuclear neutrophil granulocyte (PMN), lymphocyte (LY), platelet count (PLT), and hematocrit were selected for further toxicity assessment of inorganic NPs *in vivo* and presented in Fig. 5. No obvious signs of inflammatory responses or toxic reactions that could be attributed to the inorganic NPs were found. Moreover, there were no distinct differences in hematological parameters for inorganic NPs with different intrinsic chemical compositions. Some minor changes in PLT were observed in the NPs treated groups, which was in agreement with recent studies using nanosilicas^39^ and ZnS and ZnO QDs^40^ and CdSe/ZnS QDs^41^; however, they were within the normal range that had been reported in the literature. Other biochemical and hematology data were included in the Supplementary information (Figs S1 and S2).

### Histology results

**Figure 6:**
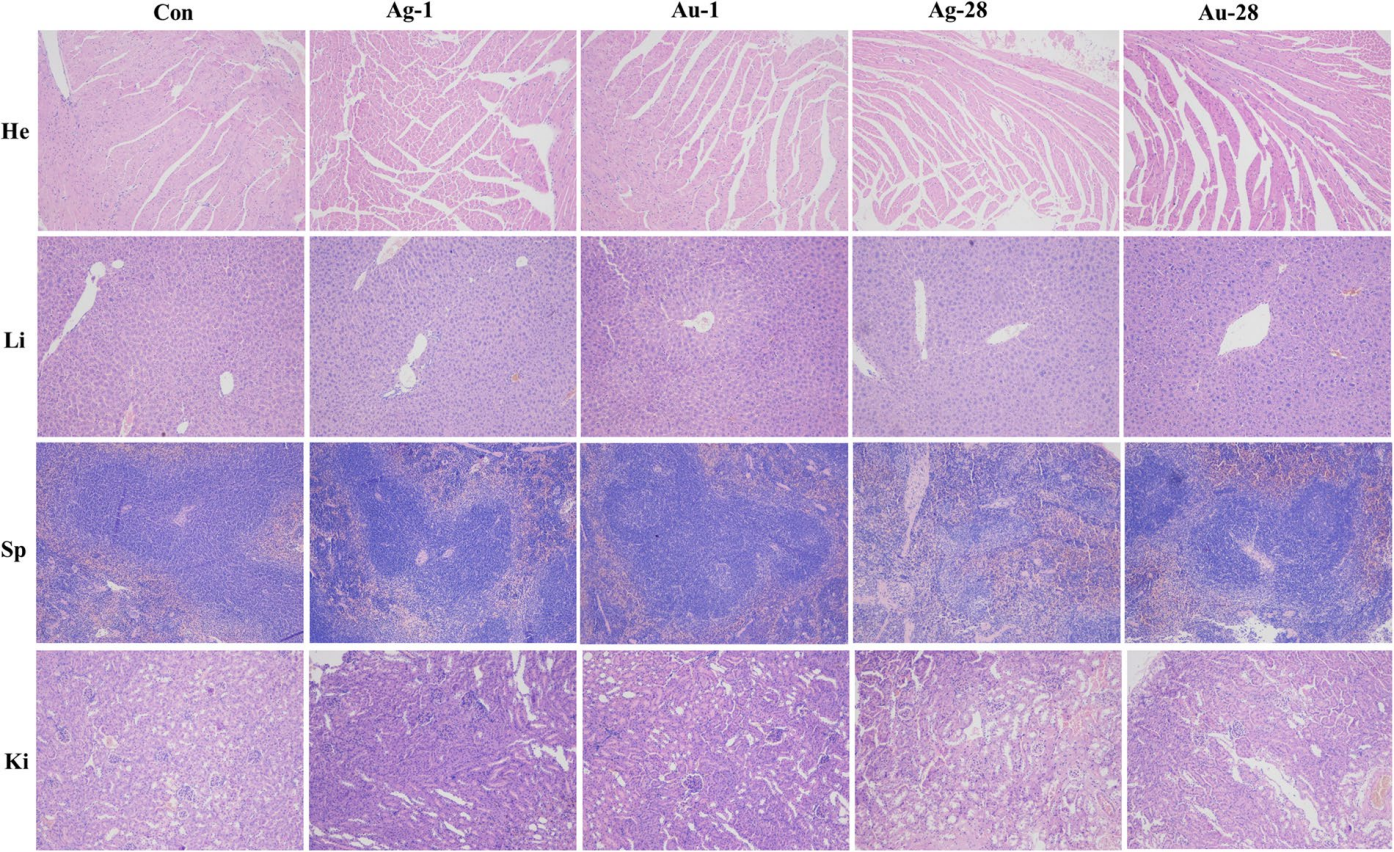
Histological images in treated animals exhibit no signs of toxicity. Heart (He), liver (Li), spleen (Sp), and kidney (Ki), from animals treated with AgNPs or AuNPs and control. Ag-1, Ag-28, Au-1, Au-28 represent the organs were collected from mice at 1 and 28 dpi, respectively. n = 5.

Histopathological examination of organs to determine potential NPs-induced tissue damage, inflammation, or lesions from exposure were performed. As shown in Fig. 6 and S3, no apparent histopathological abnormalities or lesions were observed from the animals treated with the AgNPs and/or AuNPs in comparison with the control. These findings demonstrated that AgNPs and/or AuNPs at dosage of 11.4–13.3 mg/kg revealed insignificant pathological changes.

### Gene expression profiles

**Figure 7:**
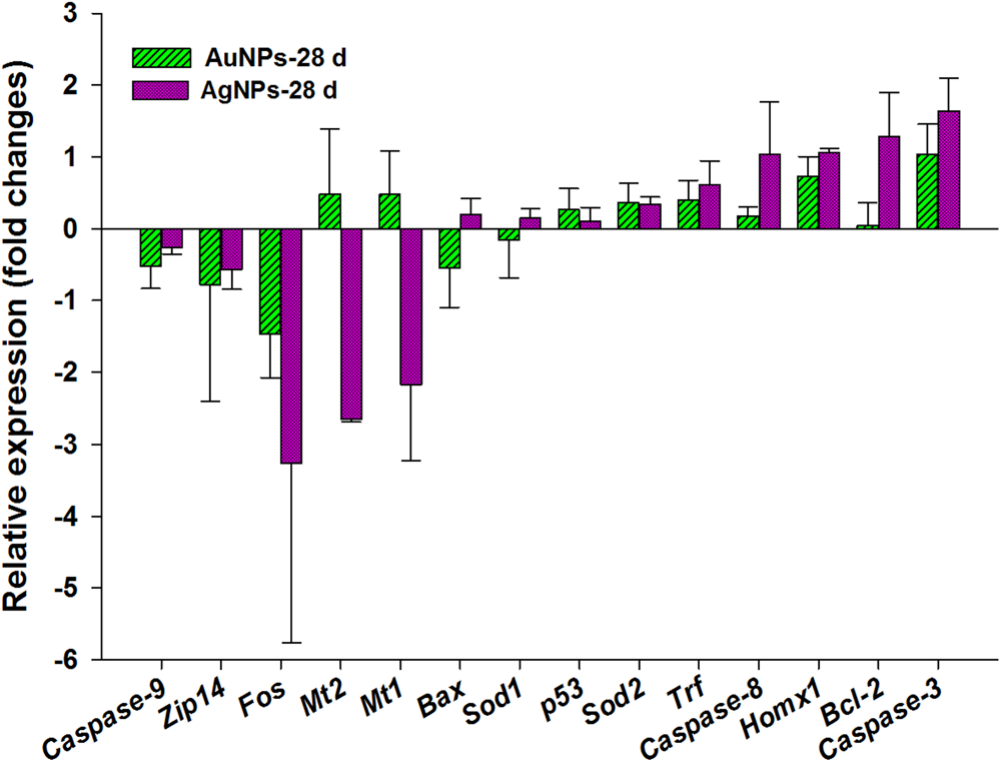
Gene expression changes in the liver of mice treated with AgNPs, AuNPs, and vehicle group on 28 dpi. The relative expression ratio refers to fold changes in the histogram which a ratio greater than zero such as 0.5 indicated gene expression increased by 0.5 time, whereas a ratio below zero indicated downregulation.

The transcriptional levels of genes with functions associated to cell apoptosis, ion transport, and oxidative stress were further determined to understand the subtle differential damages of AgNPs and AuNPs in liver deposited highest amount of NPs in this study. As presented in Fig. 7, exposure to AgNPs and AuNPs induced mild and similar gene expression changes with downregulation of 3 genes expression such as *Caspase-9*, V-fos FBJ murine osteosarcoma viral oncogene homolog (*Fos*), and Zrt- and Irt-related protein 14 (*Zip14*) and upregulation of 7 genes expression including *p53*, superoxide dismutase 2 (*Sod2*), Heme oxygenase 1 (*Hmox1*), *Caspase-3*, *Caspase-8*, transferrin (*Trf*), and *Bcl-2* in total 14 genes investigated in this study. In particular, *Fos* was down-regulated to 1.17–3.25 fold changes, whereas *Homx1* and *Caspase-3* were up-regulated from 0.73 to 1.64 times in the both NPs groups. Additionally, the residual genes used here had an opposite gene expression. For instance, mRNA levels of Metallothionein 1 (*Mt1*) and Metallothionein 2 (*Mt2*) were increased to approximately 0.48 fold change in AuNPs group, while their levels were declined to 2.17–2.64 times in AgNPs normalized to that in control group. Of note, the AgNPs treatment group exhibited the greater effect in most of gene expression compared to that in AuNPs group.

## Discussion

We aimed to distinguish the biodistribution and toxicity differences between AgNPs and AuNPs by examining the body weight, organ index, organ distribution, hematology and biochemistry, histology, and gene expression profiles. First, we observed a mass decrease in spleen, brain and testis but no reduction of body weight after intravenous administration of 11.4–13.3 mg/kg AgNPs and/or AuNPs in mice. The body and organ weight proved to be susceptible to inorganic NPs intravenous exposure^11, 28, 40^. Zhang *et al*.^11^ demonstrated that 5, 10 and 45 mg/kg AgNPs caused a decrease in the body weight. Also, the liver and spleen mass increased while the thymus showed weight decrease with exposure to low doses of AgNPs (<6 mg/kg)^28^. However, the body weight and organ index were insignificantly affected by oral and inhalation exposures to both NPs^3, 12, 23, 42^. Increases in brain weight and in liver weight were observed only at doses up to 300 mg/kg per day in a 28 d oral toxicity study^20^.

The majority of AuNPs and AgNPs were accumulated in the liver and spleen, which could due to uptake by the resident phagocytes *e*.*g*. Kupffer cells in the liver and the macrophages and B cells in the spleen that are part of the MPS^13, 21, 27^. A relatively small amount of AuNPs or AgNPs were accumulated in the kidney, lungs, heart, stomach and intestines. AgNPs and AuNPs levels showed lower levels in the liver and small increments in the kidney and spleen at 28 dpi, indicating possible translocation of AgNPs and AuNPs *in vivo*. Of note, this translocation led to more accumulations in the testis and brain over time, implying that 3 ± 1.57 nm AgNPs and 6 nm AuNPs could effectively cross the blood-testis barrier (BTB) and the blood-brain barrier (BBB). The AgNPs with 28 oral administration was not cleared out of the brain and testis during a 4-month recovery period, which was consistent with our results, albeit over a shorter period of time^14^. Nevertheless, the total recovery ratios of administrated NPs were less than 50% in all groups, indicating that the residual amount of both NPs could be mainly stored in the carcass. Previous studies showed that AgNPs concentrations in the urine were extremely low compared with the concentrations in the feces^37, 43^. NPs with sizes <5.5 nm exhibited rapid and efficient urinary excretion, while 10–20 nm NPs were trapped in the liver and eliminated in the feces^44^. Renal excretion of AgNPs and AuNPs with sizes of 12.22 ± 6.05 and 15.07 ± 6.68 nm respectively could not be the primary route of elimination. Furthermore, a small number of AgNPs and even fewer AuNPs were detected in the feces, suggesting that the AgNPs but not the AuNPs were excreted through the biliary pathway from the liver to the bile duct, intestine, and feces^37^.

The difference in HD of the AgNPs and AuNPs (12.22 ± 6.05 and 15.07 ± 6.68 nm) could show minimal effects on the distribution and toxicity of NPs because AuNPs with HD of 11 nm and 21 nm showed no significant differences in terms of accumulations in the liver, spleen, lung, and kidney as well as in terms of body clearance from feces and urine at 24 h after administration^25^; and also NPs with approximate HD of 10–20 nm had a similar elimination behavior *in vivo* ^44^. Previous studies suggested that a complex protein corona is formed immediately when NPs were incubated in a biological fluid, and the surface coating controlled the composition and structure of the protein corona which was relevant to the pathophysiology *in vivo* ^45–47^. In this study, the identical surface coating (amphiphilic polymer coating with a reactive group of carboxylic acid) used for both NPs was selected to exclude the impact of surface physicochemical properties during the formation of the dynamic protein corona. The difference in distribution and circulation of AgNPs and AuNPs *in vivo*, therefore, could be attributed to the intrinsic properties of the NPs that lead to the formation of diverse protein coronas on the surface of AgNPs and AuNPs, rather than the size of the NPs. Additionally, in model zebrafish embryos, Bar-Ilan *et al*.^34^ demonstrated that AgNPs induced more severe lethal and sub-lethal effects than AuNPs at the same concentrations and sizes, implying that the NPs chemical compositions play a vital role in toxicity induction. The intravenously administered AgNPs were stored primarily in the liver and spleen, which then released silver ions that migrated and accumulated in the kidney, lung, and brain^30^. More widely accumulated AgNPs in mice that were observed in our study were therefore probably due to the released silver ions that easily translocated into organs.

Evaluation of genotoxicity is an ideal tool to assess the biosafety at molecular level for metal NPs without induction of apparent histologic lesions. The hypothesis was that susceptive genes related to oxidative stress and apoptosis could be affected as proved in previous studies^10, 20^, though no obvious injuries occurred in organs of present study. *Caspase 8* and *Caspase 9* as initiator caspases are activated by death-inducing tumor necrosis family receptors and cytochrome c, respectively, which are involved in immune and apoptosis signaling. *Caspase-3* mediates mitochondrial targeted apoptotic signaling pathways which is an executor of apoptosis^48, 49^. Activation of *p53* is associated with apoptosis through regulation of *Bcl-2* and *Bax* gene expression. *Bcl-2* is a type of apoptosis inhibiting factor while *Bax* regulates the apoptosis activation factors such as cytochrome C, ratio of their expression level reflects the state of cell apoptosis. The transcription levels of these apoptosis markers revealed that gene expression was moderate regulated in both groups, while more effectively changed was observed in liver of mice treated with AgNPs. ROS is also considered to be a mechanism of genotoxicity for metal NPs, which could promote oxidative DNA damage^8, 22^. Proteins of *Sod1* and *Sod2* are capable of scavenging free superoxide radicals and *Homx1* is an ROS sensor which exhibited excellent anti-inflammatory and antioxidant properties^49^. Stable gene expression of *Sod1* and *Sod2* and small enhancement of *Hmox1* expression indicated inefficient oxidative stress was induced in the liver of mice exposure to both NPs. Moreover, degradation of metal NPs *in vivo* could influence the ion transport of essential trace metal elements^36^. *Trf* is an iron transporter and *Zip-14* is an iron and zinc transporter showed minimal effects in expression when mice exposure to NPs. However, Metallothionein (MT) is considered to be the essential indicator of toxicity caused by heavy metals and/or metal NPs as facilitating detoxification of metal and protection from oxygen free radicals^49^. *Mt1* and *Mt2* were significantly down-regulated in liver of mice treated with AgNPs as our previous study for iron oxide magnetic NPs^36^ referred to free silver ions could be released from AgNPs. Their expressions in AuNPs group showed an opposite trend with negligible upregulation manifested that AuNPs should be more inert *in vivo*.

**Figure 8:**
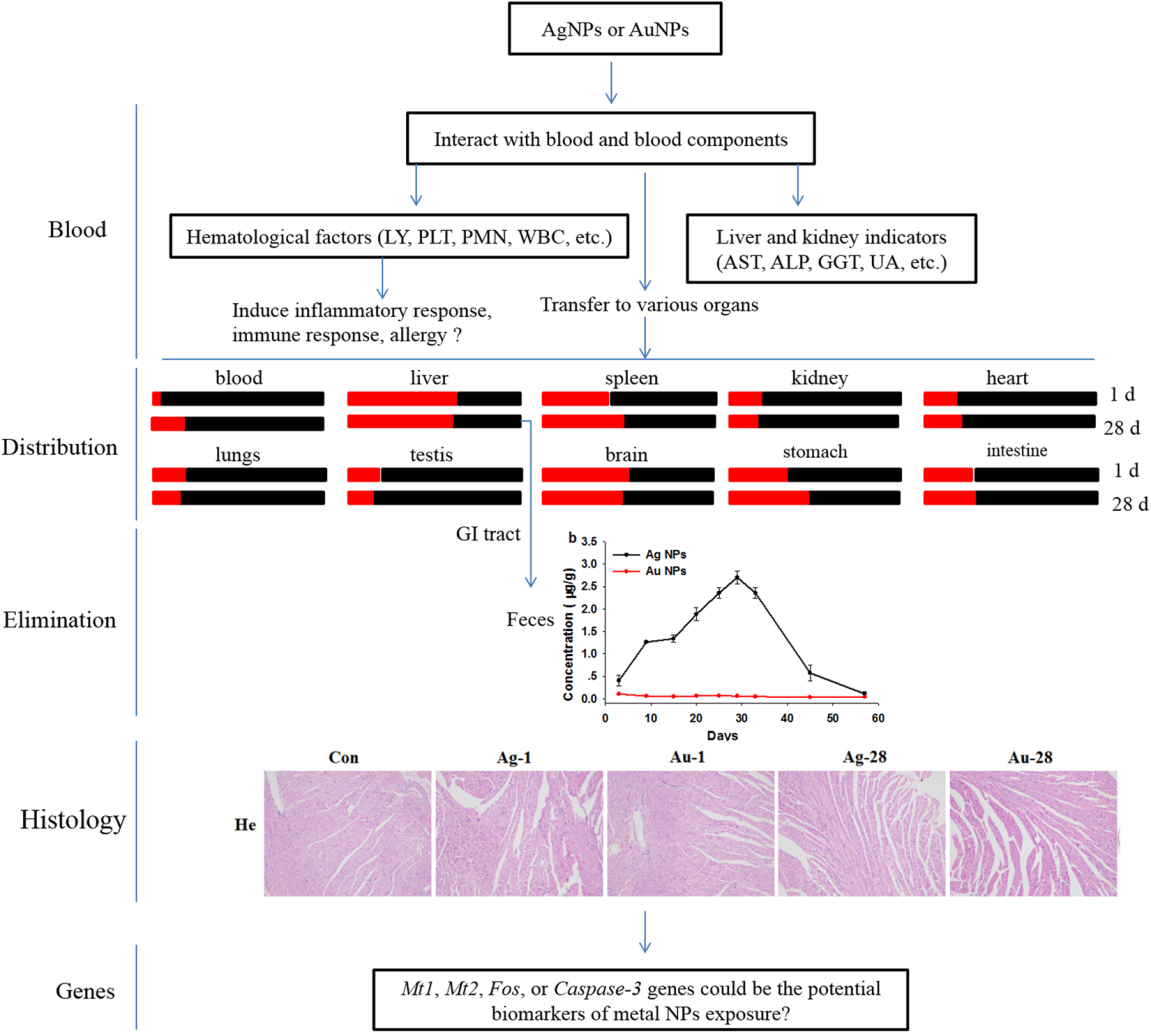
The proposed toxic and distribution difference of AgNPs and AuNPs based on this study data. The red and black boxes represent AuNPs and AgNPs treated group, respectively.  Means the ratio of the gold content over silver content in various organ.

In conclusion, the present study demonstrated that the AuNPs were mainly stored in the liver, whereas the AgNPs were widely stored in more organs including the heart, lungs, kidney, etc. The circulation of the NPs in the blood and excretions in the feces were also found to differ between the AgNPs and the AuNPs. The transcription levels of biomarkers of apoptosis, oxidative stress, and ion transport were not significantly altered except *Mt1*, *Mt2*, *Fos*, and *Caspase-3* (but still less than 3.5 fold changes normalized to control group) in livers of mice treated with both NPs on 28 dpi, while more effectively changed was observed in AgNPs group. As shown on Fig. 8, these findings suggested that NPs chemical compositions (AgNPs and AuNPs) were individually responsible for their distribution and toxic profiles. No definite signs of toxicity in mice treated with AgNPs and/or AuNPs were observed over the period of two months as indicated by observing the body weight, hematology, biochemistry, histopathology and genotoxicity. Further studies involving several apoptotic signaling pathways and other biomarkers based on transcriptomics and proteomics during long term exposure *in vivo* should be conducted to distinguish the toxicity differences caused by the chemical composition of the NPs to gather valuable information for safety.

## Methods

### Materials

Water soluble AgNPs and/or AuNPs with amphiphilic polymer coating and carboxylic acid as the reactive group used here were obtained from Ocean NanoTech, LLC (San Diego, CA). Two NPs sizes were confirmed with TEM prior to use. Briefly, NPs samples were prepared by dropping a sample onto an agar carbon-coated copper grid (400 meshes) and the solvent was evaporated. The TEM images were obtained at 50–100 K magnifications with a JEOL transmission electron microscope (JEOL USA, Inc. Peabody, MA) operating at 100 kV as previously described^36^. The HD distributions of the AgNPs and AuNPs (n = 4) and zeta potential were measured by DLS using a Zetatrac Ultra 151 (Microtrac Inc., Montgomeryville, PA).

### Experimental design

Healthy, 10–12 week old, adult male KunMing mice (30–35 g each) were purchased from the experimental animal center of Nanchang University, China. The animals were raised in an animal facility at 25 °C with a 12 h light/dark cycle; the animals were supplemented with food and water ad libitum. All procedures involving animals were approved by the Animal Care Review Committee (approval number 0064257), Nanchang University, Jiangxi, China, and were performed in accordance with the relevant guidelines and regulations of institutional animal care committee.

Thirty male mice were randomly divided into three groups (the AgNPs group, AuNPs group, and control group). Exposures were carried out via tail-vein injection with 0.1 mL of 0.5 mg/mL AgNPs or AuNPs suspension or physiological saline (control) each time, 2 times a week, for 4 weeks. The injected dosage was approximately 11.4–13.3 mg NPs/kg body weight in total, which was similar with the doses used in relevant intravenous exposure studies^19, 28^ and much less than the doses used in several oral exposure studies^3, 14, 20, 29^. The weight of the mice and food consumption were recorded throughout the entire experimental period. Collection of the blood samples and isolation of various organs were as described in our earlier study^36^. In brief, blood samples were harvested from the five mice in each group on day 1 and 28 dpi and then the mice were sacrificed. The organ samples were collected and weighed for visceral index measurement. Two samples of the organs were isolated and a small portion of tissue was immediately fixed in 10% neutral buffered formalin for histological examination. The remaining portion from each organ (0.1–0.5 g) was stored at −20 °C for elemental analysis. The organ indexes were calculated using the following eq. (1) based on a recent study^40^:

1 $${\rm{organ}}\,{\rm{indexes}}=\frac{{\rm{mass}}\,{\rm{of}}\,\mathrm{organ}\,({\rm{mg}})}{{\rm{mass}}\,{\rm{of}}\,{\rm{body}}\,\mathrm{weight}\,({\rm{g}})}$$

### Quantitative analysis of AgNPs and AuNPs in tissues

The subsamples of approximately 0.1–0.5 g tissue (including liver, spleen, kidneys, heart, lungs, brain, intestine, stomach, testis, seminal vesicle, and feces, individually) and approximately 0.3 mL whole blood were evaluated for concentrations of AgNPs or AuNPs. The subsamples were dissolved in 12 mL digestion solution (HNO_3_:HClO_4_ = 5:1) and were heated to 230 °C. The temperature was increased to 280 °C when the reaction reached equilibrium. The digested organ samples were diluted with Milli-Q water to 25 mL after removal from the heating block. For AgNPs treatment group, the digested subsamples were used to determine the silver concentrations with AAS (Thermo Scientific iCE 3500, AAS, 0.05 mg/L minimum detectable) when the concentration of AgNPs was greater than 0.05 mg/L and ICP-MS (Varian 820-MS, ICP-Mass spectrometer) when the concentration of AgNPs was less than 0.05 mg/L. With the AuNPs treatment group, the digested subsamples were used to determine the gold concentrations with ICP-OES (Optima 5300 DV, PerkinElmer) when the concentration of AuNPs was greater than 0.05 mg/L and ICP-MS when the concentration of AuNPs was less than 0.05 mg/L. The concentration of AgNPs and AuNPs in the isolated organs from the control animals was determined using ICP-MS. All sample digestions and measurements were performed by using the standard protocol established by Center of Analysis and Testing of Nanchang University, Nanchang. The contents of AgNPs and AuNPs in the different tissues were calculated using the following eq. (2):

2 $${\rm{Ag}}\,{\rm{or}}\,\mathrm{Au}\,({\rm{\mu }}g/g)\,{\rm{in}}\,{\rm{tissue}}=\frac{[{\rm{Ag}}\,{\rm{or}}\,{\rm{Au}}]\,{\rm{in}}\,{\rm{tissue}}\,{\rm{suspension}}\times 25}{{\rm{wet}}\,{\rm{weight}}\,{\rm{of}}\,{\rm{tissue}}}$$

### Blood biochemistry and hematology

Before the mice were sacrificed by cervical dislocation, blood were collected through the retro orbital plexus region in potassium EDTA collection tube and drug free tube for hematology and serum biochemistry analysis. A small amount of whole blood (0.3 mL) was collected in potassium EDTA collection tube for hematology, and approximately 0.8 mL blood was centrifuged (4000 rpm, 10 min) to obtain at least 0.25 mL blood plasma for serum biochemistry study. The residual blood of each mouse was used to determine the AgNPs or AuNPs contents. Indicators of liver and kidney functions such as ALT, AST, TBIL, ALB, A/G, GGT, ALP, BUN, and UA were monitored. WBC, RBC, HB, MCH, PMN, LY, and PLT were also determined. The whole blood with anticoagulant and the serum were examined and followed the guidelines and regulations of the First Affiliated Hospital of Nanchang University, Nanchang, China.

### Pathological examinations

A small portion of each organ was fixed in 10% neutral buffered formalin. Subsequently, isolated tissues were embedded in paraffin blocks (previously melted at 58 °C) and frozen at 4 °C before 3–5 µm sections were cut and stained with hematoxylin and eosin (H&E) for histological examination. An Olympus optical microscope (Tokyo, Japan) was used to observe the stained slices.

### RT-qPCR analysis

Since highest deposition of both NPs in the liver which was selected to the representative organ to determine the sensitive gene expression. The total RNA extraction, cDNA synthesis, and Quantitative PCR analysis were performed as our previously study^36^. In brief, RNA was extracted and cDNA was synthesized using the AxyPrepTM Multisource Total RNA Miniprep Kit (Lot#03415KD1, Case#161) and the Takara PrimeScriptTM RT reagent kit (Cat#RR047A, Lot#AK2802), respectively, according to the manufacturer’s protocol. The isolated total RNA was examined by agarose gel electrophoresis and quantified by using the NanoDrop 1000 spectrophotometer (Thermo scientific Inc.) with total RNA (1000 ng) for cDNA synthesis. Table S5 lists the qPCR primers synthesized by Invitrogen China (Shanghai, China). QPCR was carried out using SYBR® Premix Ex Taq™ II (TakaRa Code: DRR820A). The reaction mixture was prepared by mixing aliquots of cDNA, 0.8 μL (10 μM) of each primer, 5 μL SYBR® Premix Ex Taq™ II (2×) and 0.2 μL ROX Reference Dye II (50×) in a final volume of 10 μL. Amplification was performed on a 7900HT Fast Real-Time System (Applied Biosystems, Foster city, CA) with the following two-step thermal cycling program: 1 cycle at 95 °C for 30 s, then 40 cycles at 95 °C for 5 s, and then 60 °C for 1 min. Relative gene expression levels were determined by the critical threshold (Ct) number and calculated using the 2^−ΔΔCt^ method, with *GADPH* utilized as the reference gene for whole groups.

### Statistical analysis

All the data were expressed as means ± standard deviations (n = 5). Comparison of results among the groups were carried out by one-way analysis of variance (ANOVA) and L.S.D. test using SPSS v16.0 (SPSS, Inc., Chicago, IL); *P < 0.05 and **P < 0.01 were considered significant statistically and extremely significant statistically, respectively.

## Electronic supplementary material

Supplementary Information
