# Supplementary material for: Comparisons of the biodistribution and toxicological examinations after repeated intravenous administration of silver and gold nanoparticles in mice
Source: Sci Rep. 2017 Jun 12;7:3303. doi: 10.1038/s41598-017-03015-1 (PMC5468332; doi:10.1038/s41598-017-03015-1)
Supplement: Supplementary file 1 — Supplementary Information [file 41598_2017_3015_MOESM1_ESM.pdf]

Supplementary information

Comparisons of the biodistribution and toxicological examinations after repeated intravenous administration of silver and gold nanoparticles in mice

Lin Yang[1], *Huijuan Kuang*[1], *Wanyi Zhang*[2], *Zoraida P. Aguilar*[3], *Hua Wei*[1], *and Hengyi Xu*[1]*

[1]State Key Laboratory of Food Science and Technology, Nanchang University, Nanchang 330047, China

[2] The Second Affiliated Hospital of Nanchang University, Nanchang 330000, China

[3] Zystein, LLC., Fayetteville, AR 72704, USA

*Correspondence to:

Dr. Hengyi Xu

State Key Laboratory of Food Science and Technology, Nanchang University.

Address: 235 Nanjing East Road, Nanchang 330047, P.R. China.

Phone: +0086-791-8830-4447-ext-9520.

Fax: +0086-791-8830-4400.

E-mail: kidyxu@163.com; HengyiXu@ncu.edu.cn.

Table S1. Classical studies of biodistribution and toxicity of AuNPs/AgNPs in rodents via other exposure routes.

NPs	Size (nm)	Dose	Model	Exposure duration	Tissue accumulation (in order of quantity)	Conclusions	References
AgNPs	20, 15	90 mg kg^{-1}	Rats	Oral, 28 d<br>Re 1, 8, 56 d	St, In, Sp, Te, Li, Br, Ki, Lu, Bl, Bla, He	Oral exposure to AgNPs showed similar distribution pattern with silver salts, and no hepatotoxicity or immunotoxicity were observed	1
AgNPs	10, 25	100, 500 mg kg^{-1}	Rats	Oral, 28 d<br>Re 1, 2, 4 m	Sp, Ov, Li, Ki, Te, Br, Bl,	Tissue clearance of the accumulated NPs over 4 recovery months, which size did not affect the silver distribution.	2
AgNPs	60	30, 300, 1000 mg kg^{-1}	Rats	Oral, 28 d<br>Re 1 d	St, Ki, Li, Lu, Te, Br, Bl,	Gender-related and dose-dependent accumulation of silver content in tissues	3
AgNPs<br>Ag-acetate	14	63, 126, 252 mg kg^{-1}	Rats	Oral, 28 d<br>Re 1 d	NM	No adverse effects were observed in rats after exposure to 14 nm AgNPs at a dose up to 252 mg kg^{-1}	4
AgNPs<br>(PVP)	14 ± 4	252 mg kg^{-1}	Rats	Oral, 28 d<br>Re 1 d	Fe, In, Ki, Li, Br, Lu, Pl, Mu, Ur	Compared to AgNPs, silver acetate show higher concentrations throughout the rat body and lysosomes could be a likely target for side effects of silver.	5
AgNPs	7.9 ± 0.95	1, 10 mg kg^{-1}	Rats	Oral, once<br>Re 1, 4 d	Fe, Li, Bl, Ki, Lu, Ur,	Bioavailability of AgNPs was extreme low after oral exposure, suggesting limited toxicity.	6
AgNPs	20, 110	0.3, 3, 30 mg kg^{-1}	Mice	Oral, 3 d<br>Re 2 d	Fe, GIT, Sp, Li, Ki,	Oral administrational AgNPs at a dose of 30 mg kg^{-1} was well-tolerated in mice and NPs size or coating show minimal effects on fecal elimination	7
AgNPs<br>Ag-acetate	10, 75, 110	9, 18, 36 mg kg^{-1}	Rats	Oral, 13 w<br>Re 0.5 d	MLN, Co, Je, Il, Ki, Sp, Li, Bl, He, Ut	AgNPs predominantly deposited within cells, while Ag-acetate had an affinity for extracellular membranes	8
AgNPs	18	0.7×10^6, 1.4×10^6, 2.9×10^6 particles/cm^3	Rats	Ih, 6 h/d, 90 d	NM	Lung function changes such as the decreases in minute volume and tidal volume as well as lung inflammation were induced by prolonged inhalation exposure to AgNPs	9

AgNPs	18-19	0.6×10^6, 1.4×10^6, 3.0×10^6 particles/cm^3	Rats	Ih, 5 h/d, 5 d/w, 13 w	Lu, OB, Li, Br, Ki, Bl	Inhalation exposure of AgNPs caused the inflammatory responses in the lung and liver.	10
AuNPs (PEGylated)	11, 21, 31	0.07-0.30 mg kg^{-1}	Rats	Ins, 1 h, 1 d	Lu, Li, Sp, Ca, Ki, He, Bl, Br	The PEGylation had no effects on the translocation of AuNPs from the lungs to the circulation.	11
AuNPs	13, 105	12.8 ± 2.42, 13.7 ± 1.32 µg/m^3	Rats	Inh, 6 h/d, 5 d, Re 1, 3, 28 d	Lu, Li, Sp, Ki, Br, Te, Bl	Small NPs had significantly higher concentrations in secondary target organs that translocated from lungs compared to large NPs	12
AgNPs	15, 410	179, 167 µg/m^3	Rats	Inh, 6 h/d, 4 d, Re 1, 7 d	Lu, Li,	Size-related silver nanoparticle distribution in respiratory tract with 410 nm NPs mainly deposited in upper airways and 15 nm NPs showed alveolar accumulation.	13
AgNPs (PVP)	70	0.2, 1 mg kg^{-1}	Rats	Ins, 1 d	NM	Instillation of 1 mg kg^{-1} AgNPs caused mild pulmonary toxicity, while no side effects were observed at a dose of 0.2 mg kg^{-1}	14
AuNPs	1.4, 5, 18, 80, 200, 2.8	0.8 ± 0.1 – 34.5 ± 2.0 µg/rat	Rats	Ins, 1, 3, 24 h	Lu, Ur, Ki, Ca, Bl, Li, Sp, Ut, He, Br,	Small AuNPs with high specific surface area (SSA) was more likely cross the air-blood barrier. While the translocation of NPs to the secondary tissues was independent with the SSA.	15
AuNPs	12.5	0.32, 1.6, 3.2 mg kg^{-1}	Mice	i.p., 8 d	Sp, Li, Ki, Lu, Br, Bl	Dose-dependent accumulation pattern of NPs and no subacute physiological damage.	16

The following abbreviations are used in the Table: NM – not mentioned, Inh – inhalation exposure, Ins – instillation exposure, i.p. – intraperitoneal injection. Re – recovery days, h – hours, d– days, w– weeks, m– months, PVP – polymer polyvinylpyrrolidone, Ov – ovary, Pl – plasma, Br – brain, He – heart, Li – liver, Lu – lung, OB – olfactory bulb, Ki – kidney, In – intestine, MLN – mesenteric lymph nodes, Co – colon, Il – ileum, Je –jejunum, Sp – spleen, GIT – gastro-intestinal tract, Fe – feces, St – stomach, Te – testes, Ut – uterus, Ur – urine, Ca – carcass, Bla – bladder, Bl – blood, Mu – muscle.

References

1. Zande, M. *et al.* Distribution, elimination, and toxicity of silver nanoparticles and silver ions. *ACS Nano* **6**, 7427-7442 (2012).

2. Lee, J. H. *et al.* Biopersistence of silver nanoparticles in tissues from Sprague –Dawley rats. *Part. Fibre Toxicol.* **10**, 36 (2013).

3. Kim, Y. S. *et al.* Twenty-eight-day oral toxicity, genotoxicity, and gender-related tissue distribution of silver nanoparticles in Sprague-Dawley rats. *Inhal.Toxicol.* **20**, 575-83 (2008).

4. Sung, J. H. *et al.* Subacute oral toxicity investigation of nanoparticulate and ionic silver in rats. *Arch. Toxicol.* **86**, 543-51 (2012).

5. Loeschner, K. *et al.* Distribution of silver in rats following 28 days of repeated oral exposure to silver nanoparticles or silver acetate. *Part. Fibre Toxicol.* **8**, 18 (2011).

6. Park, K. *et al.* Bioavailability and toxicokinetics of citrate-coated silver nanoparticles in rats. *Arch. Pharm. Res.* **34**, 153-58 (2011).

7. Bergin, I. L., *et al.* Effects of particle size and coating on toxicologic parameters, fecal elimination kinetics and tissue distribution of acutely ingested silver nanoparticles in a mouse model. *Nanotoxicology* **10**, 352-360 (2016).

8. Boudreau, M. D., *et al*. Differential Effects of Silver Nanoparticles and Silver Ions on Tissue Accumulation, Distribution, and Toxicity in the Sprague Dawley Rat Following Daily Oral Gavage Administration for 13-Weeks. *Toxicol. Sci.* kfv318 (2016)

9. Sung, J. H. *et al.* Lung function changes in Sprague-Dawley rats after prolonged inhalation exposure to silver nanoparticles. *Inhal.Toxicol.* **20**, 567-74 (2008).

10. Sung, J. H. *et al.* Subchronic inhalation toxicity of silver nanoparticles. *Toxicol.Sci.* **108**, 452-61 (2009).

11. Lipka, J. *et al.* Biodistribution of PEG-modified gold nanoparticles following intratracheal instillation and intravenous injection. *Biomaterials* **31**,6574-81 (2010).

12. Han, S. G., *et al.* Size-dependent clearance of gold nanoparticles from lungs of Sprague–Dawley rats after short-term inhalation exposure. *Arch. Toxicol.* **89**, 1083-1094 (2015).

13. Braakhuis, H. M., *et al.* Particle size dependent deposition and pulmonary inflammation after short-term inhalation of silver nanoparticles. *Part. Fibre Toxicol.* **11**, 1 (2014).

14 Haberl, N., *et al*. Cytotoxic and proinflammatory effects of PVP-coated silver nanoparticles after intratracheal instillation in rats. *Beilstein J. Nanotech.* **4**, 933-940 (2013).

15 Kreyling, W. G., *et al*. Air–blood barrier translocation of tracheally instilled gold nanoparticles inversely depends on particle size. *ACS nano* **8**, 222-233 (2013).

16. Lasagna-Reeves, C. et al. Bioaccumulation and toxicity of gold nanoparticles after repeated administration in mice. *Biochem. Bioph. Res. Co.* **393**, 649-55 (2010).

Table S2. The visceral index of organs of Kunming mice following intravenous injection of AgNPs and/or AuNPs. D1 and D28 refer to collection of mice organ on post-injection in day 1 and day 28. These results show mean and standard deviation, n=5. *: P < 0.05 versus the control group.

NPs	BW (g)	Liver (mg/g)	Spleen (mg/g)	Kidney (mg/g)	Lung (mg/g)	Heart (mg/g)	Brain (mg/g)	Testis (mg/g)	Seminal vesicle (mg/g)
AgNPs (D1)	38.59±1.81	54.11±3.22	5.67±1.75	14.86±1.44	5.96±0.59	4.87±0.68	11.26±0.52	6.20±1.12*	7.59±0.84
AuNPs (D1)	39.33±3.06	46.59±6.94	4.65±1.25	14.92±1.65	6.25±1.59	5.99±1.08	10.25±0.74	5.90±0.63*	7.71±1.97
AgNPs (D28)	42.65±1.68	50.68±9.70	2.58±0.36*	14.67±3.49	6.68±1.73	5.38±0.72	9.04±0.52*	5.73±0.84*	6.20±2.13*
AuNPs (D28)	42.87±3.18	49.46±5.66	2.95±0.91*	16.18±1.59	6.91±2.61	5.43±0.69	9.62±1.68	6.23±0.94*	5.78±0.63*
Control	41.16±1.07	52.42±4.92	4.77±1.06	18.08±0.61	7.54±2.73	6.44±1.13	11.18±0.25	8.18±1.39	8.87±1.47

Table S3. Biodistribution of Ag and/or Au NPs at 1 d and 28 d after the last injection. These results show mean and standard deviation, n=5.

IOMNs	Heart (μg/g)	Liver (μg/g)	Spleen (μg/g)	Lung (μg/g)	Kidney (μg/g)	Brain (μg/g)	Stomach (μg/g)	Intestine (μg/g)	Testis (μg/g)	Seminal vesicle (μg/g)
AgNPs (D1)	5.00 ± 0.94	35.06±4.78	21.16±11.74	4.64±0.23	3.43±1.15	0.65±0.07	1.14±0.21	1.93±0.37	3.72±0.29	0.38±0.11
AuNPs (D1)	1.18±0.14	61.39 ± 19.99	12.98±1.03	1.06±0.23	5.27±0.72	0.69±0.04	0.60±0.08	0.75±0.18	0.82±0.18	0.18±0.01
AgNPs (D28)	3.85 ± 1.10	29.68±4.50	24.78±6.72	5.03±1.06	0.83±0.14	0.82±0.08	0.65±0.13	2.02±0.83	5.85±1.46	0.30±0.07
AuNPs (D28)	1.03±0.19	47.00±10.62	22.03±2.30	0.90±0.09	1.01±0.14	0.71±0.03	0.55±0.17	0.84±0.16	0.98±0.02	0.20±0.05
Control (AgNPs)	ND	ND	ND	ND	0.01±0.003	ND	ND	ND	ND	ND
Control (AuNPs)	0.04±0.02	0.02±0.03	0.04±0.03	0.031±0.002	0.015±0.0003	0.02±0.0001	0.016±0.0002	0.02±0.0005	0.02±0.005	0.03±0.0017

ND: not detected

Table S4. Recovery ratios of total applied doses of AgNPs and/or AuNPs in both time points. These results show mean and standard deviation, n=5.

	**Recovery Ratios of applied doses**
AgNPs (D1)	35.87± 9.94%
AuNPs (D1)	31.17± 9.67%
AgNPs (D28)	42.54± 18.87%
AuNPs (D28)	27.78± 6.13%

Table S5. The primes used in qPCR.

Genes	Descriptions	Forward primers (5'—3')	Reverse primes (5'—3')
Bax	Bcl2-associated X protein (Bax)	GATGGCAACTTCAACTGGG	CCGAAGTAGGAGAGGAGGC
Bcl-2	*Bcl-2*	CACTCGACCTTGTTTCTTCCAG	TCCTAACCCCTTGCTCTGCTT
Caspase-8	*Caspase-8*	GGAAGATGACTTGAGCCTGCTTG	CAAGGCTCATTCTTCTCTCTGTGC
Caspase-3	*Caspase-3*	GGAGGCTGACTTCCTGTATGCTT	CCTGTTAACGCGAGTGAGAATG
Caspase-9	*Caspase-9*	AAGAAGACCGGAGTGCAATG	CATGACAGGATTATACAACCGC
p53	*p53*	TACAAGAAGTCACAGCAC	GATACTCGGGATACAAAT
Mt2	Metallothionein 2	TGCATCTGCAAAGAGGCTTC	AAGTTGTGGAGAACGGGTCAG
Mt1	Metallothionein 1	CTGCTGCTCCTGCTGTC	ACTGTATAGGAAGACGCTGG
Zip14	Zrt- and Irt-related protein 14	CATTGAAGTATGGGGGTACGGT	ATGAAGTAGAGCAGGAGCCTCT
Trf	Transferrin	CCGAACAACAAAGAGGAAT	GGTTCTTTCCTTCGGTGTT
Hmox	Heme oxygenase 1	ACCGCCTTCCTGCTCAAC	GAGGAGCGGTGTCTGGGAT
Sod 1	superoxide dismutase 1	TAACTGAAGGCCAGCATGGGT	GGTCTCCAACATGCCTCTCTTC
Sod 2	superoxide dismutase 2	CAGACCTGCCTTACGACTATGG	GCTGAAGAGCGACCTGAGTTGT
Fos	V-fos FBJ murine osteosarcoma viral oncogene homolog	GAAGGGAAAGGAATAAGATGGC	AGTTGGTCTGTCTCCGCTTG
GADPH	Glyceraldehyde-3-phosphate dehydrogenase	ACCCAGAGGACTGTGGATGG	TCAGCTCTGGGATGACCTTG

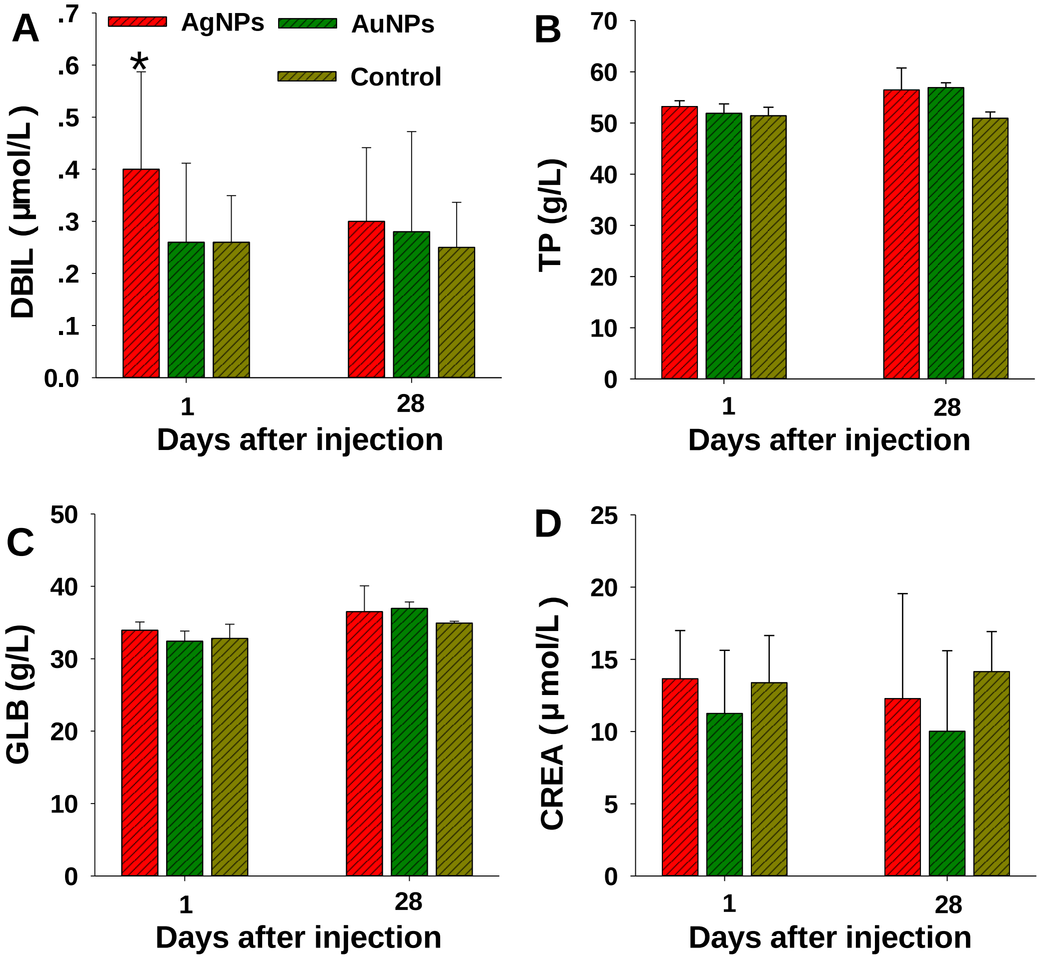

Fig. S1 Serum biochemical analysis from animals treated with AgNPs and/or AuNPs and control. A-D) results exhibit mean and standard deviation of DBIL (A), TP (B), GLB (C), CREA (D). Abbreviations: direct bilirubin, DBIL; total protein, TP; globulin, GLB; creatinine, CRE. n=5. *: $P < 0.05$ versus the control group.

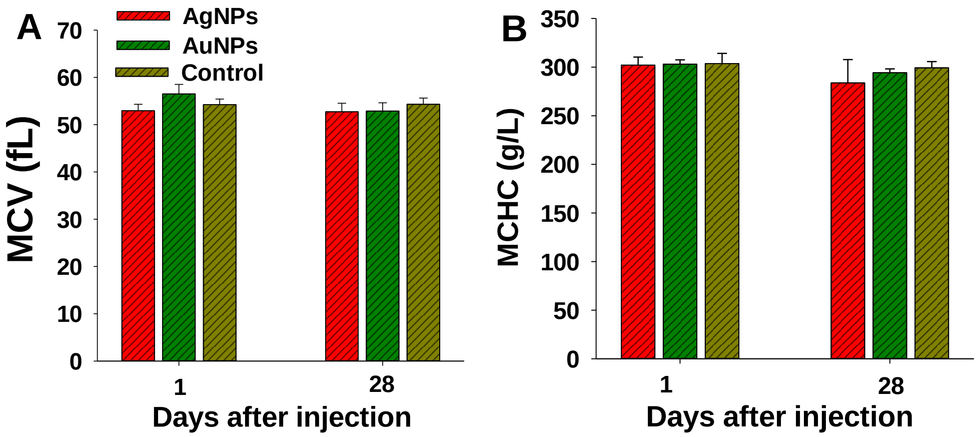

Fig. S2 Whole blood analysis from animals treated with AgNPs and/or AuNPs and control. A,B results are based on mean and standard deviation of mean corpuscular volume (MCV) and mean corpuscular hemoglobin concentration (MCHC), respectively. These indicators are not significant changes ($p < 0.05$), n=5.

Heart Liver

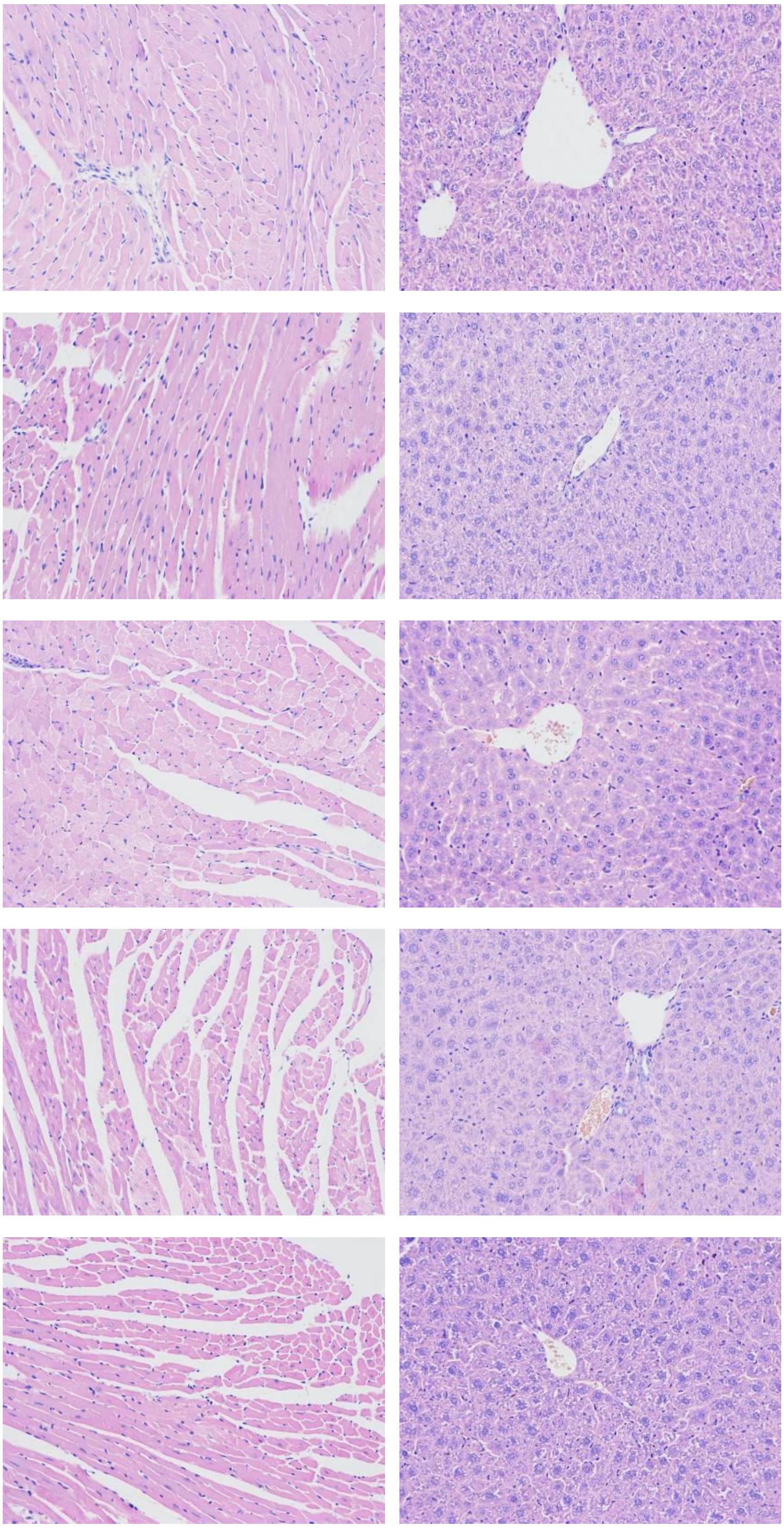

Control

AgNPs (D1)

AuNPs (D1)

AgNPs (D28)

AgNPs (D28)

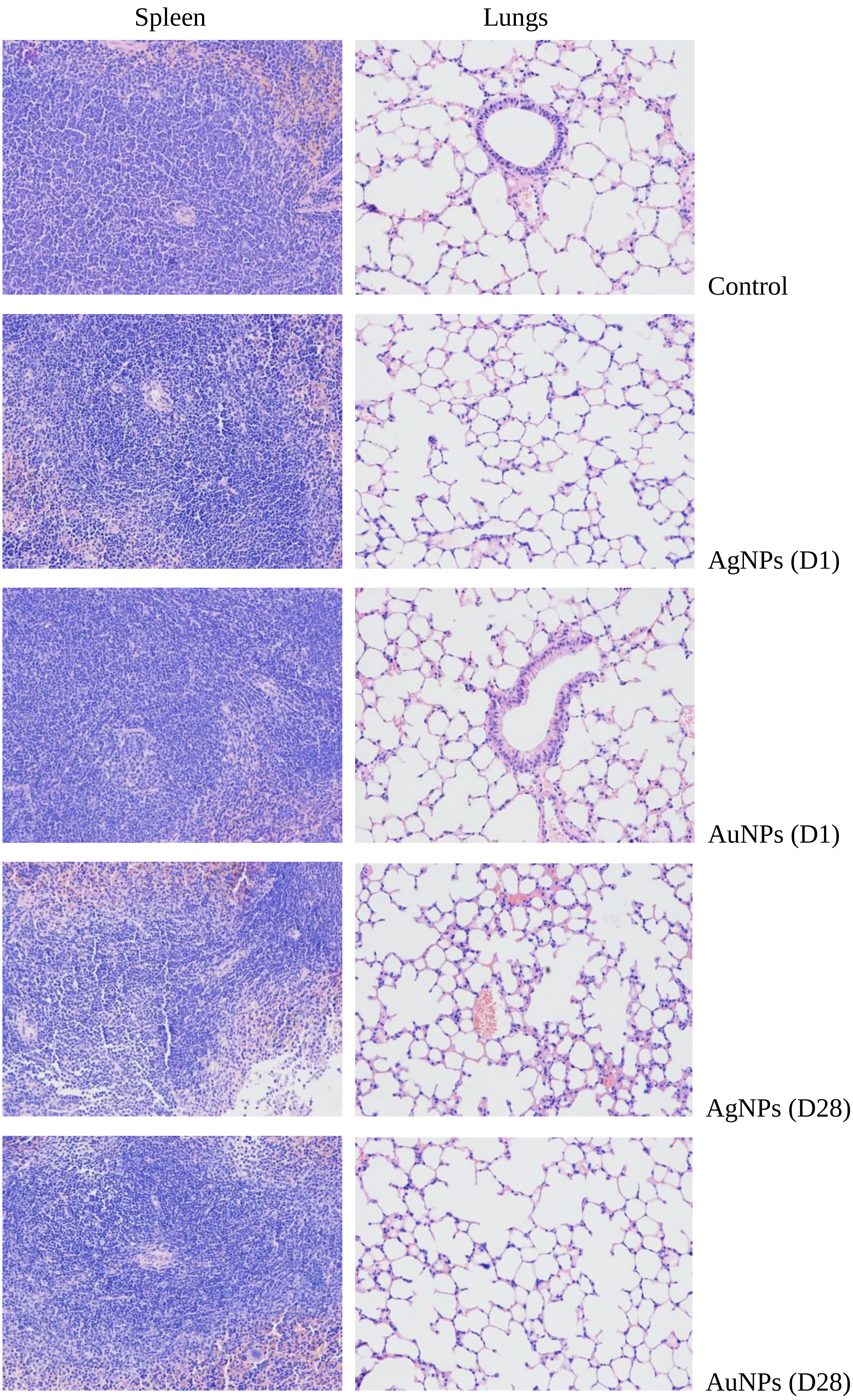
Spleen
Lungs
Control
AgNPs (D1)
AuNPs (D1)
AgNPs (D28)
AuNPs (D28)

Kidney Brain

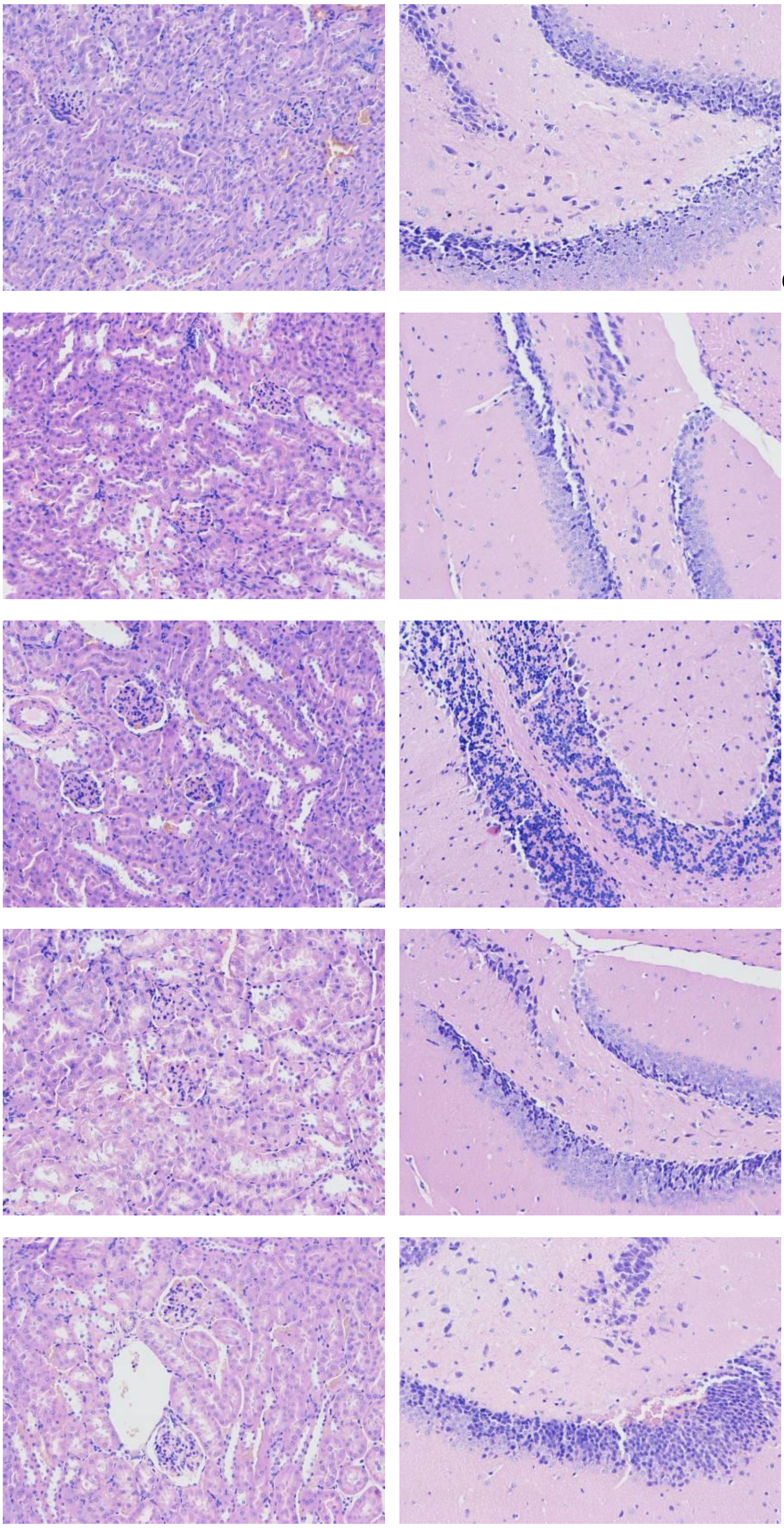

Control

AgNPs (D1)

AuNPs (D1)

AgNPs (D28)

AuNPs (D28)

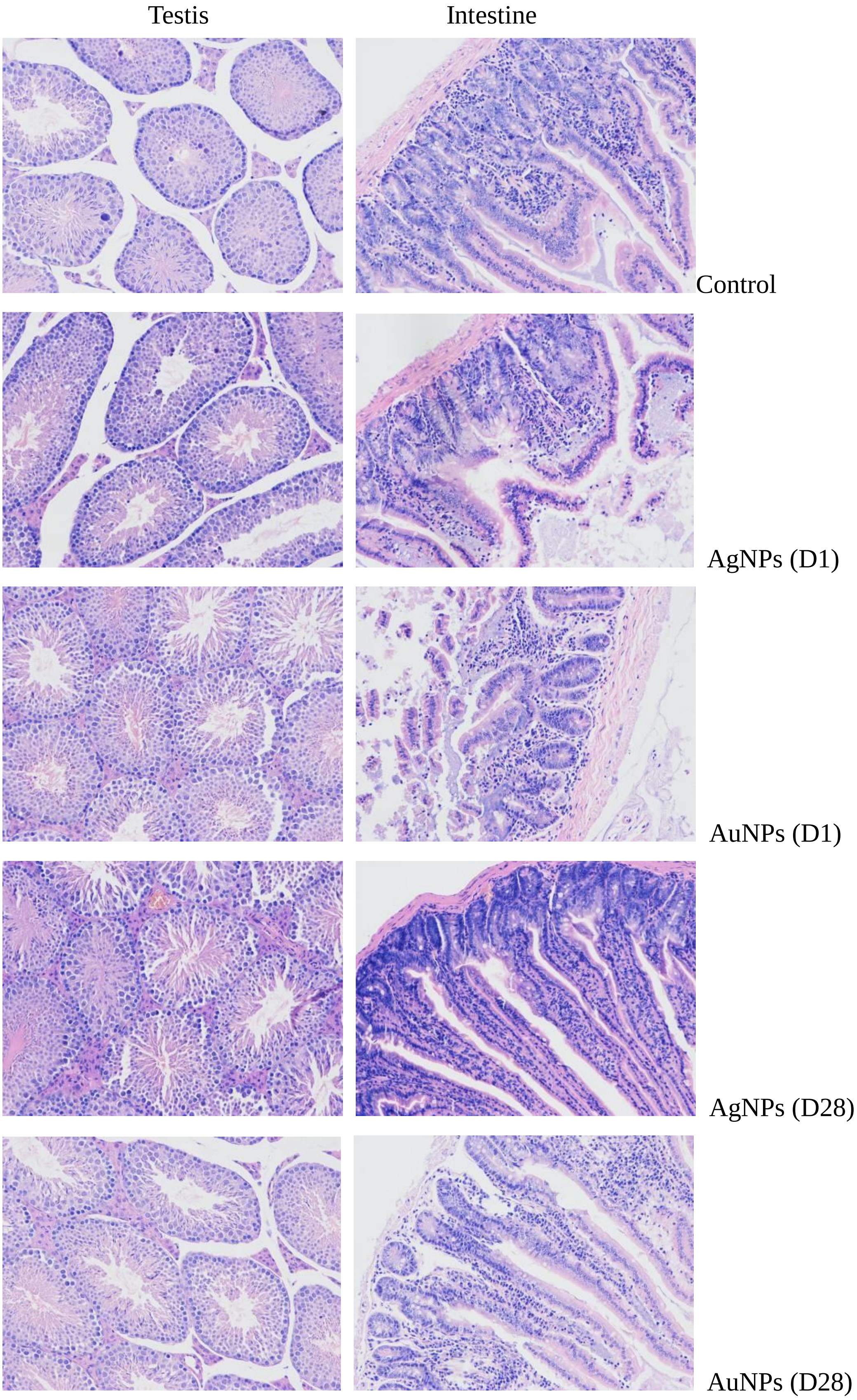

Fig. S3 HE images of various organs of treated AgNPs and/or AuNPs mice. D1 and D28 refer to collection of mice organ on day 1 and day 28 after AgNPs and/or AuNPs administrations, respectively. n=5.
